# Quality of Kangaroo Mother Care services in Ethiopia: Implications for policy and practice

**DOI:** 10.1371/journal.pone.0225258

**Published:** 2019-11-22

**Authors:** Haftom Gebrehiwot Weldearegay, Araya Abrha Medhanyie, Mulugeta Woldu Abrha, Lisanu Tadesse, Ephrem Tekle, Bereket Yakob, Tsinuel Girma, Catherine Arsenault

**Affiliations:** 1 College of Health Sciences, Mekelle University, Mekelle, Ethiopia; 2 Tigray Health Research Institute, Mekelle, Ethiopia; 3 JSI, L10K and Federal Ministry of Health, Addis Ababa, Ethiopia; 4 Maternal and Child Health Directorate, Federal Ministry of Health, Addis Ababa, Ethiopia; 5 Department of Global Health and Population, Harvard T.H. Chan School of Public Health, Boston, Massachusetts, United States of America; Western Sydney University, AUSTRALIA

## Abstract

**Background:**

Providing high-quality kangaroo mother care (KMC) is a strategy proven to improve outcomes in premature babies. However, whether KMC is consistently and appropriately provided in Ethiopia is unclear. This study assesses the quality of KMC services in Ethiopia and the factors associated with its appropriate initiation among low birth weight neonates.

**Methods:**

We used data from the 2016 national Emergency Obstetric and Newborn Care (EmONC) assessment which contains data on all health facilities providing delivery care services in Ethiopia (N = 3,804). We described the quality of KMC services provided to low-birth weight (LBW) babies in terms of infrastructure, processes and outcomes (survival status at discharge). We also explored the factors associated with appropriate KMC initiation using multivariable logistic regression models.

**Results:**

The quality of KMC services in Ethiopia was poor. The facilities included scored only 59.0% on average on a basic index of service readiness. KMC was initiated for only 46.4% of all LBW babies included in the sample. Among those who received KMC, 66.7% survived, 13.3% died and 20.4% had no data on survival status at discharge. LBW babies born in health centers were twice more likely to receive KMC compared to those born in hospitals (AOR = 2.0, 95% CI: 1.3–3.0). Public facilities, those with a staff rotation policy in place for newborn care, and those with separate newborn corners were also more likely to initiate KMC for LBW babies.

**Conclusions:**

We found low levels of appropriate KMC initiation, inadequate infrastructure and staffing, and poor survival among LBW babies in Ethiopia. Efforts must be made to improve the adoption of this life saving technique, particularly in hospitals and in the private sector where KMC remains underutilized. Facilities should also dedicate specific spaces for newborn care that enables mothers to provide KMC. In addition, improving record keeping and data quality for routine health data is a priority.

## Introduction

Every year, 15 million babies are born premature and their complications result in over one million deaths [1]. Kangaroo Mother Care (KMC) is an evidence-based inpatient care technique for premature and low-birth weight (LBW) babies that is recommended for all newborns weighing less than 2000 grams [2–3]. KMC involves close follow-up of the baby’s growth and a sustained, long-lasting, skin-to-skin contact between the mother and the newborn [4]. Good-quality KMC involves the prevention and management of infections, respiratory and feeding support, thermal control, and involves post-discharge follow-up [5]. Evidence shows that KMC results in significant reductions in pain, infection, hypoglycemia, breathing problems and hospital readmissions. KMC is also associated with improved cognitive development throughout the child’s life [6–7]. Among the psychological benefits to the mother, KMC is linked to reductions in stress and increased mother-to-baby bonding [8].

According to Ethiopian and World Health Organization (WHO) guidelines, all LBW babies should receive KMC. Sick and very small babies needing special care should initially be cared for under radiant warmer and KMC should be initiated once the baby is hemodynamically stable [9–10]. Providing KMC to all LBW babies is also one of the targets of Ethiopian Health Sector Transformation Plan (HSTP) to improve newborn survival.

In spite of the evidence on its benefits, adoption of KMC in low-income countries remains limited and its implementation has faced several barriers [4–5,11]. Few data exist on the quality of care provided to LBW babies in Ethiopia and it remained unclear whether KMC was consistently provided. In this analysis, we describe the quality of KMC in Ethiopia–in terms of infrastructure, processes and outcomes–and explore the factors associated with appropriate KMC initiation. Findings from this analysis will inform policy-makers about current performance of the health system in caring for LBW babies and will serve as a starting point to improve KMC services in Ethiopia.

## Methods

### Data source

We used data from the 2016 Ethiopian Emergency Obstetrics and Newborn care (EmONC) assessment [12]. The EmONC assessment was a national cross-sectional survey of all public hospitals, health centers and private facilities (higher clinics and above) that provided maternal and newborn health services and reported attending births in the past 12 months. The EmONC assessment did not include health posts or medium and small private clinics because these facilities are not expected to attend deliveries. Of the eligible 4,385 facilities in all nine regions and two city administrations in Ethiopia, 3,804 facilities were assessed (including 293 hospitals, 3,459 health centers and 52 clinics). A total of 11 facilities were not accessible due to political unrest or staff refusal. The survey used 13 questionnaires including 12 health facility assessment modules and one health system assessment module. These were adapted from the Averting Maternal Death and Disability program [12]. The survey collected data on EmONC signal functions, facility readiness (including the availability of equipment, guidelines, human resources, infection prevention measures, etc.), volume of services and maternal and newborn outcomes [12–13]. A module on newborn complications was designed to collect information on premature babies weighing less than 2000 grams. Trained data collectors extracted information from charts identified through facility registries or from the staff. In each facility, interviewers were expected to review charts for the last three LBW babies born in the past 12 months. Data on treatments provided and survival status were extracted. In most facilities, only one LBW baby chart was reviewed.

### Measures

We measured KMC quality using the three domains of quality defined by Donabedian: infrastructure, processes and outcomes [14].

### Infrastructure quality

Structural quality was assessed using all facilities included in the national EmONC survey.

We used three indicators of service availability (facility density, maternity bed density and core health workforce density) and by one index for service readiness. These indicators were adapted from the WHO Service Availability and Readiness Assessment (SARA) manual. Facility density was calculated by the number of health facilities providing maternity care per 10,000 population. Maternity bed density was calculated by the number of maternity beds per 1,000 pregnant women and the health work force density was calculated by the number of core medical professionals per 10,000 population [11, 15–16]. The service readiness index was based the availability of a series of items necessary for the provision of maternal and newborn care including specific indicators related to KMC. The index covered four domains: infrastructure and equipment, essential medicine and commodities, core staffing and guidelines, job aids and documentation. The items and calculations are described in S1 Appendix.

### Process quality and outcomes

Process quality was assessed using a binary variable for whether KMC was initiated for each LBW baby. This is a measure of appropriate treatment and competent care [17]. Because most facilities had data on only one LBW baby, we selected only the last LBW baby per facility. Because KMC cannot be initiated until the baby is stable, we also looked at the proportion of LBW babies that were initially put in incubators. Outcomes were assessed based on the survival status at discharge for all LBW babies who received KMC. Process quality and outcomes were only assessed in the subset of facilities with data on LBW babies.

### Covariates

We selected a series of facility- and provider-level covariates that may be associated with quality of care. These were selected based on prior literature on the factors affecting health care provider performance and quality in low income countries [17]. Facility characteristics included facility type (hospitals or maternal and child health (MCH) specialty centers, health centers and higher clinics), urban location, managing authority (public or private), whether the facility had a separate newborn corner, a newborn intensive care unit (NICU) and a policy in place for staff rotation. Facility types were based on definitions by the Federal Ministry of Health. Hospitals and MCH specialty centers generally have operating theaters while health centers and clinics do not. Provider characteristics included cadre, work experience in years, age, gender, and whether the provider had a written job description.

### Statistical analysis

Infrastructure processes and outcomes were assessed using descriptive statistics. We also looked at associations between each of the facility- and provider-level covariates and appropriate KMC initiation using bivariable logistic regressions. Covariates with a p-value of ≤0.25 were considered for inclusion in the multivariable logistic regression model with the forward likelihood ratio method. In the final multivariable model, a p-value<0.05 was used to determine statistical significance. All analyzes were performed using SPSS version 21^TM^ software.

### Research ethics

Ethical approval for the original survey was granted by the Scientific and Ethical Review Office of the Ethiopian Public Health Institute (EPHI). The Federal Ministry of Health of Ethiopia granted access to the data for this analysis. The institutional review board of Mekelle University considers this analysis as exempt from ethical review as it is a secondary analysis of de identified data.

## Results

**Table 1** shows the characteristics of all facilities included in the EmONC assessment (N = 3804). The majority of facilities included were health centers (90.9%) and only 293 facilities (7.7%) were hospitals. The largest numbers of facilities (37%) were found in Oromia, the largest region in Ethiopia. Of the 3,804 facilities surveyed by EmONC, only 768 facilities (20.2%) had data on at least one LBW baby.

**Table 1:** Characteristics of facilities included in the EmONC assessment 2016 (N = 3804).

Regions	Hospitals (all types) N (%)	Health centers N (%)	Higher clinics N (%)	Total N
**Tigray**	39(15.3)	213(83.5)	3(1.2)	255
**Afar**	5(6.5)	71(92.2)	1(1.3)	77
**Amhara**	59(6.7)	811(92.6)	6(0.7)	876
**Oromia**	75(5.3)	1,322(94.1)	8(0.6)	1,405
**Ethio-Somali**	11(6.8)	146(90.7)	4(2.5)	161
**Benishangul-Gumuz**	3(7.0)	40(93.0)	0	43
**SNNP**	61(7.9)	708(91.6)	4(0.5)	773
**Gambella**	1(3.7)	26(96.3)	0	27
**Harari**	6(40.0)	8(53.3)	1(6.7)	15
**Addis Ababa**	34(22.5)	92(60.9)	25(16.6)	151
**Dire Dawa**	6(28.6)	15(71.4)	0	21
**Total**	**293(7.7)**	**3,459(90.9)**	**52(1.4)**	**3,804**
**Managing authority**				
**Government**	242(6.6)	3,417(93.3)	3(0.1)	3,662 (96.0)
**Private**	58(40.8)	35(24.6)	49(34.5)	142 (0.04)
**Total**	**300(7.9)**	**3452(90.8)**	**51(1.3)**	**3,804**

We found poor service availability and readiness in Ethiopia. We estimated a facility density of only 0.41 facilities per 10,000 people, 6.68 maternity beds per 1,000 pregnant women, and 7.51 core health workers per 10,000 people in Ethiopia **[Table 2].** Comparing these results to the recommended WHO targets, we found that the three service availability indices represented only between 20.7% and 66.8% of the recommended targets.

**Table 2:** Kangaroo mother care service availability in Ethiopia, EmONC assessment 2016.

	**Number of facilities providing deliveries**	**Total population** ^**a**^	**Result**	**WHO target**	**Proportion of target achieved**
**Facility density**	3,804	92,085,000	0.41 per 10,000	2 per 10,000	20.7%
	**Number of maternity beds** ^**b**^	**Nb of expected pregnancies** ^**c**^	**Result**	**WHO target**	**Proportion of target achieved**
**Maternity bed density**	21,186	3,172,455	6.68 per 1,000	10 per 1,000	66.8%
	**Number of providers** ^**d**^	**Total population** ^**a**^	**Result**	**WHO target**	**Proportion of target achieved**
**Core health workforce density**	69,153	92,085,000	7.51 per 10,000	23 per 10,000	32.7%

a Total population is from EmONC report and is based on reported facility catchment population

b Number of beds dedicated specifically for antenatal, labor and delivery, abortion or postpartum care.

c Number of expected pregnancies is from Health and Health related indicators EFY 2009, Federal Ministry of Health

d Includes MDs, OBGYN, pediatrician, neonatologists, midwives, nurses and health officers.

**Fig 1:**
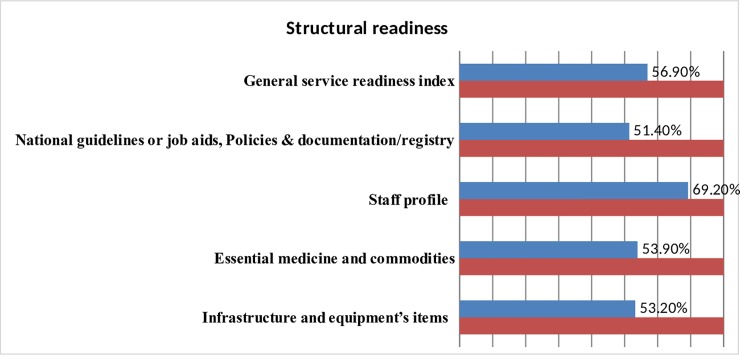
KMC service readiness index in Ethiopia, BEmONC Assessment 2016.

Facilities scored only 59.4% on average for service readiness Domain-specific readiness scores were 53.2% for infrastructure and equipment, 60.3% for availability of essential medicine & commodities, 69.2% for core staff profile, and 54.8% for availability of national guidelines or job aids and documentation **(Fig 1 and S1 Appendix)**.

Of the 768 LBW babies included in this analysis, only 356 (46.4%) received KMC **[Table 3]**. A total of 102 (13.3%) were put in incubators, and 368 (47.9%) LBW babies received neither service. In health centers and clinics, 49.0% of LBW babies received KMC compared to 38.8% in hospitals. Nonetheless, in hospitals 43.9% of LBW babies were put in incubators compared to only 2.6% in health centers or clinics. LBW babies were more likely to receive neither service in health centers compared to hospitals. Despite small sample sizes, we looked at differences in quality across regions. LBW babies were more likely to receive KMC in Tigray and Amhara, and least likely in Addis Ababa. However, Addis Ababa had the highest proportion of babies who were put in incubators.

**Table 3:** KMC process quality and outcomes by facility characteristics, EmONC assessment 2016.

Variables	KMC initiation N (%)		Survival status of LBW babies who received KMC N (%)		
	Yes	No	Alive	Died	No information
**Overall**	356(46.4)	412(53.6)	237(66.7)	46(12.9)	73(20.4)
**Region**					
Tigray	69(67.9)	33(32.4)	57(82.6)	5(7.2)	7(10.1)
Afar	4(44.4)	5(55.6)	4(100.0)	0	0
Amhara	118(56.8)	90(43.3)	79(66.9)	14(11.9)	25(21.2)
Oromia	94(36.4)	116(63.6)	60(63.8)	13(13.8)	21(22.3)
Ethio-Somali	5(35.7)	9(64.3)	3(60.0)	2(40.0)	0
Benishangul-Gumuz	2(33.3)	4(66.7)	1(50.0)	1(50.0)	0
SNNP	45(43.7)	58(56.3)	29(64.4)	9(20.0)	7(15.6)
Gambella	4(50.0)	4(50.0)	3(75.0)	1(25.0)	0
Harari	2(33.3)	4(66.7)	2(100.0)	0	0
Dire Dawa	6(40.0)	9(60.0)	4(66.7)	0	2(33.3)
Addis Ababa	7(17.9)	32(82.1)	6(85.7)	1(14.3)	0
**Facility type**					
Hospital /MCH centers	76(38.8)	120(61.2)	57(75.0)	10(13.2)	9(11.8)
Health center /higher clinic	280(49.0)	292(51.0)	191(68.2)	36(12.9)	53(18.9)
**Facility location**					
Urban	188(45.6)	224(54.4)	125(66.5)	27(14.4)	36(19.1)
Rural	168(47.2)	188(52.8)	123(73.2)	19(11.3)	26(15.5)
**Managing authority**					
Government	350(48.2)	376(51.8)	243(69.4)	45(12.9)	62(17.7)
Private	6(14.3)	36(85.7)	5(83.3)	1(16.7)	0

Of those who received KMC, 66.7% survived, 12.9% died, and survival status at discharge was missing in 20.4% of cases. Survival status among babies who received KMC was highest in Harari followed by Dire Dawa and Addis Ababa. Survival status was missing in 18.9% of health centers and clinics, compared to 11.8% of hospitals. Government facilities were more likely to initiate KMC (48.2%) compared to privately owned facilities (14.3%). The proportion of LBW babies who received KMC and survived was 6.8 percentage-points higher in hospitals compared to health centers. Survival was also higher in private (83.3%) compared to government owned facilities (69.4%). **Table 4** shows the characteristics of facilities where LBW babies were born and of the providers who assisted these deliveries.

**Table 4:** Characteristics of facilities and providers who delivered low-birthweight babies (N = 768), EmONC assessment 2016.

Variable	N	Percentage %
**Facility type**		
Hospital /MCH centers	196	25.5
Health center /higher clinic	572	74.5
**Managing authority**		
Government/Public	726	94.5
Private	42	5.5
**Facility has a separate newborn corner**		
Yes	281	36.6
No	487	63.4
**Facility has a separate NICU**		
Yes	487	63.4
No	133	17.3
**Facility has a staff rotation policy for maternal care**		
Yes	304	39.6
No	464	60.4
**Facility has a staff rotation policy for newborn care**		
Yes	293	38.2
No	475	61.8
**Provider cadre**		
MD/Health officer	15	2.0
Midwife	697	90.8
Nurse	56	7.2
**Work experience in years**		
< 2	153	19.9
2–5	497	64.7
> 5	118	15.4
**Age in years**		
< 25	424	55.3
25–40	323	42.0
> 40	21	2.7
**Gender**		
Female	480	62.5
Male	288	37.5
**Written job description**		
Yes	218	28.4
No	549	71.6
**Provider had training on completing registers and compiling reports**		
Yes	211	27.5
No	556	72.5

In bivariable analysis, only four of the 13 variables tested were associated with KMC initiation with p-values<0.25 [**Table 5]**. These were uniquely at the facility level and were included in the multivariable model. In the adjusted model, we found that facilities with separate newborn corners were more likely to initiate KMC services compared to their counterpart (AOR = 1.49, 95% CI: 1.06–2.10). Facilities that had a policy in place for staff rotation in newborn care (more than one rotation a year) were 1.84 times more likely to initiate KMC compared to facilities without staff rotation policies (AOR = 1.84, 95% CI: 1.34–2.51). Similarly, health centers were twice more likely to initiate KMC compared to hospitals (AOR = 2.0, 95% CI: 1.34–3.00). Private facilities were also less likely to provide KMC compared to government-owned facilities (AOR = 0.21, 95% CI: 0.05–0.68).

**Table 5:** Bivariable and multivariable logistic regressions for the association between facility- and provider-level characteristics and KMC initiation, EmONC assessment 2016 (N = 768).

Characteristics	Crude OR (95% CI)	Adjusted OR (95%CI)
**Provider cadre**		
MD/HO	Ref	
Midwife	0.75(0.27–2.09)	
Nurse	0.76(0.24–2.37)	
**Provider experience in years**		
<2	1.29(0.79–2.11)	
2–5	1.60(1.06–2.42)	
>5	Ref	
**Age of provider in years**		
≤ 25	0.70(0.29–1.70)	
25–40	0.56(0.23–1.37)	
>40	Ref	
**Gender**		
Female	0.75(0.56–0.99)	
Male	Ref	
**Facility has a separate newborn corner**		
Yes	0.78(0.57–0.93)	**1.49 (1.06–2.10)**
No	Ref	Ref
**Facility has a separate NICU**		
Yes	1.06(0.73–1.54)	
No	Ref	
**Facility has a staff rotation policy for maternal care**		
Yes	0.63(0.47–0.84)	
No	Ref	
**Facility has a staff rotation policy for newborn care**		
Yes	0.57 (0.43–0.77)	**1.84 (1.34–2.53)**
No	Ref	Ref
**Facility type**		
Health center /higher clinic	1.51(1.09–2.11)	**2.00 (1.34–3.00)**
Hospital /MCH centers	Ref	Ref
**Facility location**		
Urban	Ref	
Rural	1.07(0.80–1.42)	
**Facility managing authority**		
Private	0.18(0.07–0.43)	**0.21 (0.05–0.68)**
Government	Ref	Ref
**Provider has a copy of job description**		
Yes	Ref	
No	1.55(0.85–2.82)	
**Provider had training on completing registers and compiling reports**		
Yes	Ref	
No	1.18(0.85–1.62)	

## Discussion

In this analysis, we used data from a national health facility survey to describe the quality of KMC services in Ethiopia and investigate potential factors associated with appropriate KMC initiation. We found poor quality infrastructure, low KMC initiation (only 46.4% of eligible LBW babies received KMC) and poor survival among those who received KMC (only 67% were alive at discharge). Health centers, government-owned facilities, those with separate newborn corners and those with a staff rotation policy for newborn care were more likely to initiate KMC. We also found that record keeping was poor and the survival status at discharge was missing for one fifth of the LBW baby charts reviewed.

These findings have implications for developing strategies to improve LBW baby survival. Although KMC is a relatively inexpensive service, basic infrastructure, equipment, medical supplies and human resources are needed to ensure its adoption [18]. We found very low service availability and readiness for KMC services in Ethiopia. For example, although the WHO recommends a minimum of 23 health care providers per 10,000 people in low-income countries, core health workforce density was only 7.5 per 10,000 [16]. Among the factors associated KMC initiation, we found that facilities with a separate newborn corner and those with a staff rotation policy for newborn care were more likely to initiate KMC. These findings reflect the need for better infrastructure and better staffing and training to facilitate KMC. Other studies had similar conclusions. A systematic review found that lack of private space for mothers to perform KMC and to remain in the health facility with the newborn hindered KMC uptake [9]. Staffing shortages and poor staffing support also hindered providers’ ability to supervise KMC and provide mothers with the necessary counseling [9, 19–21]. KMC is a complex intervention with multiple elements that requires careful follow-up and monitoring by health care providers to identify potential newborn complications and manage them promptly [9, 11, 20, 22]. Dedicating adequate space for KMC and improving staffing and training on KMC is necessary to improve LBW babies’ outcomes in Ethiopia.

Despite KMC being a target of Ethiopia’s HSTP, we found that only 46.4% of eligible babies received KMC [4]. Health centers were twice more likely to initiate KMC compared hospitals, and public facilities were also more likely to initiate KMC compared to private facilities. This result differs from other studies done in Malawi and South Africa that found higher KMC utilization in hospitals compared to lower-level facilities [19, 23]. In Ethiopia, LBW babies born in hospitals were more likely to be put in incubators. Women who end up delivering in hospitals are more likely to suffer from complications and thus the baby may be too unstable and the mother may be too sick to provide KMC immediately after birth [24]. However, KMC should be provided once the baby becomes stable. Our study identified a need to promote the use of KMC in hospitals in Ethiopia among stable LBW babies. Similarly, providers in public facilities may be more likely to comply with national guidance on KMC compared to those in private health facilities who receive less oversight from the public health sector. KMC is a service that is free of charge and this might dissuade private facility providers from promoting its use [25–26]. Nonetheless, private sector engagement will be essential for wider reach and acceptance of the technique.

Only two thirds of LBW babies who received KMC were alive at discharge. This high mortality rate among newborns who received the technique may reflect inadequate supervision and follow-up and that the KMC is not implemented well. When adequately provided, KMC has been shown to substantially reduce the risk of neonatal mortality among LBW babies [3].

Finally, we found that many facilities failed to properly maintain registers and patient charts. Record keeping was particularly problematic for recording newborn survival status. Up to a fifth of LBW babies were missing data on survival status at discharge. Missing survival status was more common in health centers and clinics compared to hospitals. Improving the accuracy of routine health data and improving record keeping should be prioritized.

This study has several limitations. First, our measure of KMC process quality was limited to whether KMC was initiated. The EmONC survey did not contain additional information on the specific clinical actions undertaken by providers, the length of follow up, the quality of counseling and other elements of quality. In addition, the assessment was based on data extracted from charts and KMC registers that are often incomplete. Nonetheless, our study is one of the first to assess KMC services at the national level in Ethiopia. Quality of KMC was also assessed using several indicators of infrastructure, processes and outcomes that provide a more detailed picture of the state of KMC services. In addition, our study is limited to LBW babies born in health facilities. At the time of the survey, skilled delivery care was only 26% in Ethiopia. The majorities of LBW babies was therefore born at home and are unlikely to have received KMC.

## Conclusion

Important work remains to achieve universal adoption of KMC for all LBW newborns in Ethiopia. Our study provided useful information for practitioners and policy makers. Our study highlighted the importance of dedicating specific spaces for KMC in health facilities, ensuring adequate staffing and scheduling for providers to adequately supervise KMC. We also identified a need to promote KMC in private facilities and in hospitals. LBW babies born in hospitals were often cared for under radiant warmer but were less likely to receive KMC once stable. These recommendations serve as a starting point to improve KMC in Ethiopia. Future research should explore the quality of KMC using observations of care and client exit interviews.

## Supporting information

S1 Appendix Structural readiness of health facilities For KMC service in Ethiopia, EmONC 2016. (DOCX)

## Abbreviations

EmONC: Emergency obstetrics and newborn care
KMC: Kangaroo-mother care
LBW: Low birth weight
FMOH: Federal ministry of health
HSTP: Health sector transformation plan
