# Supplementary material for: Quality of Kangaroo Mother Care services in Ethiopia: Implications for policy and practice
Source: PLoS One. 2019 Nov 22;14(11):e0225258. doi: 10.1371/journal.pone.0225258 (PMC6874352; doi:10.1371/journal.pone.0225258)
Supplement: S1 Appendix — (DOCX) [file pone.0225258.s001.docx]

|  | | | | **Structural Readiness** | | |
| --- | --- | --- | --- | --- | --- | --- |
|  |  |  |  | **KMC service** | **Number of Facilities** | **N (%)** |
| **Infrastructure and equipment’s items** | | | | Designated space or beds for KMC | 3804 | 630 (16.6) |
|  |  |  |  | Functional electricity at maternity room | 3804 | 326(84.9) |
|  |  |  |  | Water source at maternity room | 3804 | 253(65.9) |
|  |  |  |  | Newborn anatomical model(for practice) | 3804 | 431(11.3) |
|  |  |  |  | Means of ventilation (e.g. window) | 3804 | 3191(83.9) |
|  |  |  |  | Functioning toilet for patient use | 3804 | 3032(79.7) |
|  |  |  |  | Radiant warmer | 3804 | 1425(37.5) |
|  |  |  |  | Incubator | 3804 | 413(10.9) |
|  |  |  |  | Baby weight scale | 3804 | 3737(98.2) |
|  |  |  |  | Cord tie | 3804 | 3622(95.5) |
|  |  |  |  | Thermometer for newborn | 3804 | 2104(55.3) |
|  |  |  |  | Caps or hats to prevent heat loss | 3804 | 877(23.1) |
|  |  |  |  | Towels/blanket or cloth for newborn | 3804 | 1106(29.1) |
| **Infrastructure and equipment’s items readiness** | | | | | | **53.2 %** |
| **Essential medicine and commodities** | | | IV fluid (neonatal giving) set | | 3804 | 1379(36.3) |
|  |  |  | Daily patient chart | | 3804 | 2024(53.2) |
|  |  |  | Syringes(0.5,1.0ml) | | 3804 | 3259(85.7) |
|  |  |  | Nasogastric feeding tube size 4 | | 3804 | 818(21.5) |
|  |  |  | Cap and spoon for infant feeding | | 3804 | 1384(36.4) |
|  |  |  | Cap for breast milk expression | | 3804 | 1261(33.2) |
|  |  |  | Oxygen source | | 3804 | 586(15.4) |
|  |  |  | **Antibiotics for Newborn** | | | |
|  |  |  | Amoxicillin (injection) | | 3771 | 149(4) |
|  |  |  | Ampicillin (injection) | | 3771 | 2611(69.2) |
|  |  |  | Augmentin | | 3771 | 2261(60) |
|  |  |  | Cefotaxime injection (for newborn) | | 3771 | 254(6.7) |
|  |  |  | Oral flucloxacillin (for newborn) | | 3771 | 345(9.1) |
|  |  |  | Gentamicin (injection) | | 3771 | 2595(68.8) |
|  |  |  | Tetracycline eye ointment/drops | | 3771 | 3068(81.4) |
|  |  |  | Option B+regimen for newborn | | 2040 | 1673(82) |
|  |  |  | Oral rehydration solution | | 3780 | 3167(83.8) |
|  |  |  | Vitamin K (for newborn) | | 3780 | 3109(81.7) |
|  |  |  | Nystatin (oral) (for newborn) | | 3780 | 208(5.5) |
|  |  |  | Polio 0 | | 3780 | 3259(86.2) |
|  |  |  | Insecticide-treated bednets (ITN) | | 3780 | 1418(37.5) |
|  |  |  | BCG vaccine | | 3780 | 3534(93.5) |
|  |  |  | Chlorehexidine (4% gel for cord cleansing) | | 3780 | 510(13.5) |
|  | | | **Infection Prevention Basic Items:** | | | |
|  |  |  | Soap | | 3802 | 3286(86.4) |
|  |  |  | Antiseptic | | 3802 | 3547(93.3) |
|  |  |  | Disposable latex gloves | | 3802 | 3690(97.1) |
|  |  |  | Heavy duty gloves | | 3802 | 3097(81.5) |
|  |  |  | Eye shields | | 3802 | 2517(66.2) |
|  |  |  | Mask | | 3802 | 2892(76.1) |
|  |  |  | Non-sterile protective clothing | | 3802 | 3244(85.3) |
|  |  |  | Decontamination container | | 3802 | 3603(94.8) |
|  |  |  | Bleach or bleaching powder(Chlorine) | | 3802 | 3210(84.4) |
|  |  |  | prepared disinfection solution | | 3802 | 3460(91) |
|  |  |  | Regular trash bin | | 3802 | 3232(85) |
|  |  |  | Covered contaminated trash bin | | 3802 | 2373(62.4) |
|  |  |  | Puncture-Proof sharps container | | 3802 | 3664(96.4) |
|  |  |  | **Disinfectants and antiseptics:** | | | |
|  | | | Disinfectant and antiseptics-Chlorehexidine (4&) gel | | 3802 | 566(14.9) |
|  |  |  | Chlorehexidine solution (savalon) | | 3802 | 3176(83.5) |
|  |  |  | Ethanol 75% | | 3802 | 1662(43.7) |
|  |  |  | Ethanol 95% | | 3802 | 568(14.9) |
|  |  |  | Polyvidone iodine | | 3802 | 2967(78) |
|  |  |  | Alcohol-based rub | | 3802 | 2759(72.6) |
| **Essential medicine and commodities readiness** | | | | |  | **60.3%** |
| **Core staff profile** | | Trained on KMC | | | 3800 | 2912(76.6) |
|  |  | Trained on Essential newborn care | | | 3800 | 3075(80.9) |
|  |  | Trained on antenatal corticosteroids for women at risk of preterm birth | | | 3800 | 1298(34.2) |
|  |  | Trained in to begin IV fluids | | | 3800 | 3236(85.2) |
| **Core staffing readiness** | | | | |  | **69.2%** |
| **National guidelines or job aids & documentation** | Guideline/protocol for KMC | | | | 3802 | 1812(47.7) |
|  | KMC register complete and up-to-date | | | | 3804 | 564(14.8) |
|  | Discharge register form | | | | 288 | 136(47.2) |
|  | Maternal newborn death report form | | | | 3804 | 1389(36.5) |
|  | **Small/sick newborn care: does the facility have:** | | | | | |
|  | Audit or carry out maternal death review in a routine basis? | | | | 3800 | 1157(30.4) |
|  | Implemented MDSR initiative | | | | 3800 | 2608(68.6) |
|  | MDSR committee established? | | | | 2608 | 2426(93) |
|  | Routine newborn death/SB audit Yes for both | | | | 3800 | 1353(35.6) |
|  | Routine neonatal death only | | | | 3800 | 116(3.1) |
|  | Routine near miss review | | | | 3800 | 1099(28.9) |
|  | System in place regularly to collects MNH service data? | | | | 3803 | 3674(96.6) |
|  | Compile any report containing MNH services? | | | | 3674 | 3666(99.8) |
|  | Designed data manager responsible for services | | | | 3674 | 2565(69.8) |
|  | Management protocol for selected emergency obstetric newborn care | | | | 3802 | 1987(52.3) |
|  | Facility provide neonatal care 24hrs a day ,7days a week | | | | 3798 | 3708(97.6) |
| **National guidelines or job aids & documentation readiness** | | | | | | **54.8%** |
| **KMC service readiness index** | | | | | | **59.4%** |
